# Supplementary figures and images for: Location of Recurrences after Trimodality Treatment for Glioblastoma with Respect to the Delivered Radiation Dose Distribution and Its Influence on Prognosis
Source: Cancers (Basel). 2023 May 30;15(11):2982. doi: 10.3390/cancers15112982 (PMC10252044; doi:10.3390/cancers15112982)

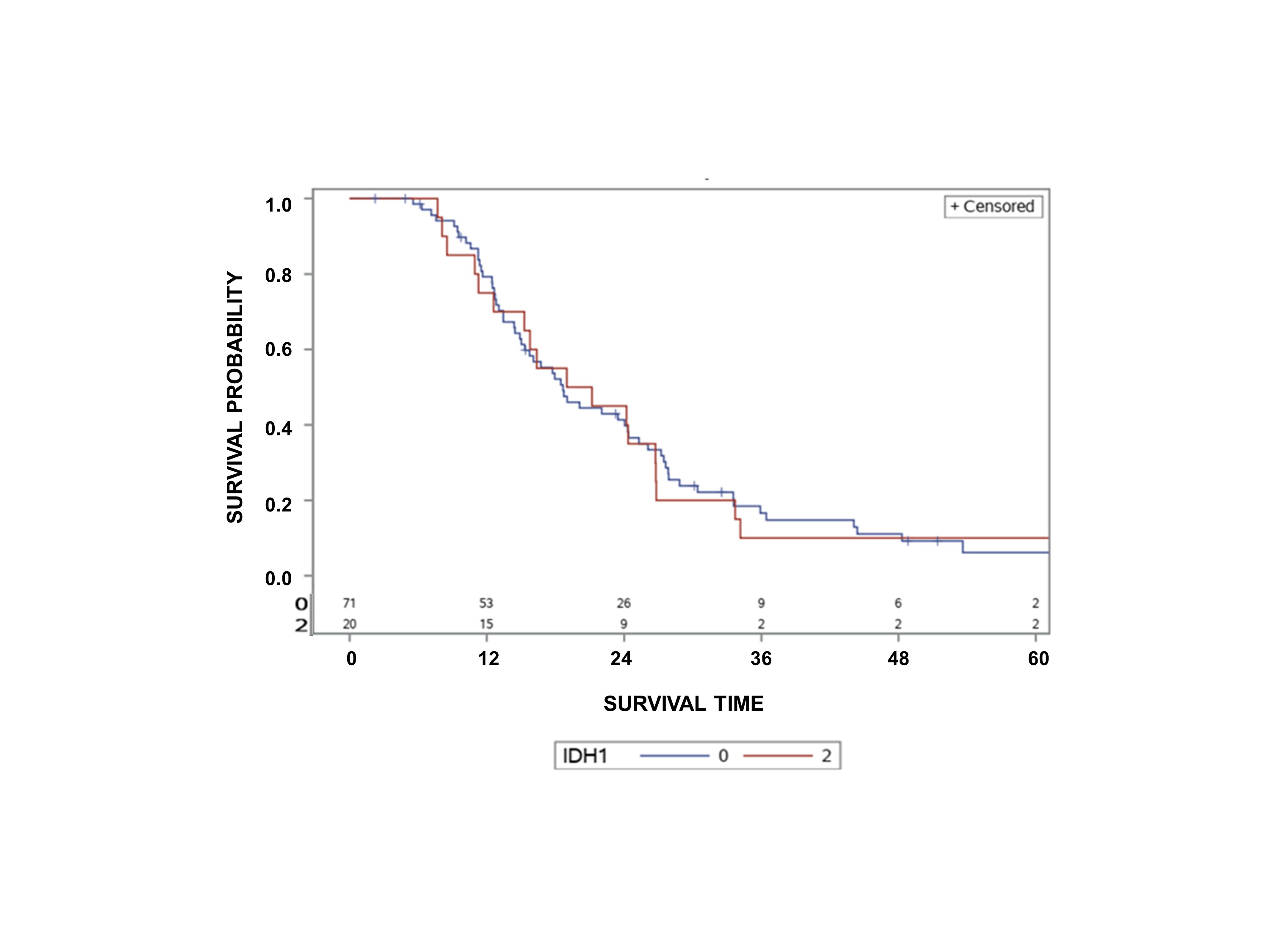

Supplement: Supplementary file 1 [file cancers-15-02982-s001.zip › cancers-2332904-Figure S1.jpg]
